# Protocol for a Systematic Review of Telehealth Privacy and Security Research to Identify Best Practices

**DOI:** 10.5195/ijt.2015.6186

**Published:** 2015-11-20

**Authors:** VALERIE J.M. WATZLAF, DILHARI R. DEALMEIDA, LEMING ZHOU, LINDA M. HARTMAN

**Affiliations:** 1 DEPARTMENT OF HEALTH INFORMATION MANAGEMENT, SCHOOL OF HEALTH AND REHABILITATION SCIENCES, UNIVERSITY OF PITTSBURGH, PITTSBURGH, PA, USA; 2 HEALTH SCIENCES LIBRARY SYSTEM, UNIVERSITY OF PITTSBURGH, PITTSBURGH, PA, USA

**Keywords:** Privacy, protocol, security, systematic review, telehealth, telerehabilitation

## Abstract

Healthcare professionals engaged in telehealth are faced with complex US federal regulations (e.g., HIPAA/HITECH) and could benefit from the guidance provided by best practices in Privacy and Security (P&S). This article describes a systematic review protocol to address this need. The protocol described herein uses the Preferred Reporting Items for Systematic Review and Meta-Analysis Protocols (PRISMA-P). The PRISMA-P contains 17 items that are considered essential, as well as minimum components to include in systematic reviews. PICOS (participants, interventions, comparisons, outcome(s) and study design of the systematic review) are also relevant to the development of best practices in P&S in telehealth systems. A systematic process can best determine what information should be included and how this information should be retrieved, condensed, analyzed, organized, and disseminated.

Healthcare professionals engaged in telehealth are faced with complex US federal regulations (e.g., HIPAA/HITECH). This article describes a systematic review protocol to identify best practices in the literature, in Privacy and Security (P & S) for telehealth.

There are many organizations that similarly define telehealth. These include the American Health Information Management Association (AHIMA), the American Telemedicine Association (ATA), Centers for Medicare and Medicaid Services (CMS), the Health Resources and Services Administration (HRSA) and the World Health Organization (WHO). HRSA’s definition most closely aligns the purposes of this article, to describe a protocol for the systematic review of research articles. HRSA (2015) defines telehealth as the use of electronic information and telecommunications technologies to support long-distance clinical health care, patient and professional health-related education, public health and health administration. Technologies include videoconferencing, the Internet, store-and-forward imaging, streaming media, and terrestrial and wireless communications (HRSA, 2015).

According to the Office of the National Coordinator for Health Information Technology, (ONC), telehealth is different from telemedicine because it refers to a *broader scope of remote healthcare services* than telemedicine. Telehealth can refer to both remote clinical services, and remote non-clinical services such as provider training, administrative meetings, and continuing medical education (Health IT.gov, 2014).

Telehealth services are commonly delivered as outpatient service to community-based homes or residential facilities. Telehealth differs from telemedicine which is normally conducted between hospitals/providers and includes services that are more acute, intense, of short duration, and with limited client involvement. The location (community, homes), the degree of consumer involvement (personal, situational), and the potentially long duration can render the Privacy & Security (P&S) issues even more challenging (Parmanto & Saptano, 2008). Telerehabilitation, the sub-set of telehealth conducted by rehabilitation specialists, is often conducted over many weeks or months.

Our experiences in interacting with telehealth providers suggest that the providers do not always know the best practices to decrease the risk of P&S issues in telehealth (Cohn & Watzlaf, 2012; Peterson & Watzlaf, 2015; Watzlaf, 2010; Watzlaf et al, 2011). Most of the P&S features within the free, consumer-based video and voice communication systems that we evaluated did not instill confidence that the information was private and secure (Watzlaf & Ondich, 2012). Nor did the telehealth providers possess strategies to best educate colleagues and consumers on P&S (Watzlaf et al., 2010, 2011).

For telehealth services to be successful, all types of healthcare information, especially sensitive personal health information that is transmitted over the Internet via videoconferencing systems, mobile health systems and store and forward apps, should engender patient confidence and trust that the information will be kept private and secure (Garg & Brewer, 2011; Hall & McGraw, 2014). Vendor systems are evolving over time and some systems have published materials stating that their systems are HIPAA compliant. Providers can use P&S protocols to determine if a system actually supports HIPAA compliance.

The Health Insurance Portability and Accountability Act (HIPAA) *Privacy rule* is an administrative law created by the Department of Health and Human Services (DHHS) that went into effect in 2003 (United States Department of Health and Human Services [UDHHS], 2003). While the *Privacy rule* only applies to healthcare providers that conduct electronic billing transactions, the rule applies to both paper and electronic health information. The HIPAA *Security rule* regulates only electronic health information and went into effect in 2005 (United States Department of Health and Human Services, 2003). The Health Information Technology for Economic and Clinical Health Act (HITECH) (HealthIT.gov, 2009) includes changes to the HIPAA Privacy and Security rules that focus mainly on health information technology and strengthens standards for the privacy and security of health information. While HITECH went into effect in 2010, some parts of the act have different compliance deadlines (Rinehart-Thompson, 2013)

Our previous work in P&S demonstrated a useful P&S checklist for providers to employ when assessing consumer-based Voice over Internet Protocol (VoIP) services. The 58-question checklist is specific to this type of Information and Communication Technologies (ICTs) (Watzlaf et al, 2010). Even though some consumer-based VoIP services do not comply with the HIPAA Privacy and Security Rule and are not recommended for use, many healthcare providers still use them because of low cost, convenience, and accessibility (Armfielda, Bradforda, & Bradforda, 2015; Sirintrapun & Cimic, 2012). Moreover, while some systems have strong encryption for their VoIP, they will not take part in a business associate agreement and do not advocate using these systems for health care services.

Our long-term objectives are to:

1. Evaluate P&S measures and HIPAA compliance in all types of telehealth services. This aim will be achieved by evaluating the technologies used in published papers in telehealth and the P&S measures used in the studies.
2. Review the literature for a clear, step-by-step approach on P&S compliance between clinician and patient when using telehealth services.
3. Compile best practices and guidelines that will prove helpful for healthcare professionals when using telehealth technologies and when educating consumers on their questions and concerns related to P&S issues.

As a first step, this article will describe a review protocol to examine telehealth research articles and identify the current P&S best practices. The primary audience for this review will include health care professionals who use telehealth technologies. The ultimate goal is to establish a recommended approach for best practices in HIPAA compliance in telehealth systems for all healthcare professionals providing telehealth services.

## METHODS/DESIGN

### PROTOCOL AND REGISTRATION

The construction of this systematic review protocol is based on the Preferred Reporting Items for Systematic Review and Meta-Analysis Protocols (PRISMA-P). The PRISMA-P contains 17 items that are considered essential as well as minimum components to include in systematic reviews or meta-analyses. PRISMA-P recommends that each systematic review include detailed criteria using the PICOS (participants, interventions, comparisons, outcome(s) and study design of the systematic review) reporting system (Moher et al, 2015). Listed below are the PICOS that are to be used in this systematic review protocol design:

**Participants:** All healthcare professionals
**Interventions:** Telehealth
**Comparisons:** Traditional practice (e.g., in-person, PoT (plain old telephone).
**Outcomes:** Privacy and security best practices
**Study Design:**
  **Inclusion criteria:** Randomized and non-randomized controlled trials, pre- and post-test designs, non-experiment observational (cross-sectional, case-series, case studies) and qualitative papers that examine the benefits and other impacts of privacy and security on the use and retention of telehealth. Machine translation will be used for articles other than English and Chinese.
  **Exclusion criteria:** Non-telehealth related delivery of service research methodologies.

### TIME FRAMES

**Inclusion criteria:** Studies performed from 2003 to the present.

**Exclusion criteria:** Studies performed prior to 2003. Telehealth technologies used before this time are likely to be outdated and predated the US federal regulations for compliance. HIPAA began to address privacy in 2003 and security in 2005.

### POPULATION

**Inclusion criteria:** All healthcare professionals using any available telehealth services for their clients.

**Exclusion criteria:** Non-healthcare professionals providing services using telehealth.

### INTERVENTION/COMPARATORS

All types of interventions will be included and none will be excluded. The traditional methods of delivering health care (face-to-face, telephone, etc.) are used as comparators. Providing care via telehealth was excluded as comparators.

### OUTCOMES

**Primary outcomes:** Primary outcomes are the most important outcomes for this systematic review and include:

1. Existing solutions/best practices to P&S challenges (qualitative and quantitative)
2. Current challenges and issues regarding P&S in telehealth, HIPAA compliance.
3. Best practices in telehealth delivery related to P&S, confidentiality, integrity, availability, HIPAA, state regulations, and other federal regulation compliance such as the Federal Trade Commission (FTC), Food and Drug Administration (FDA), Federal Communications Commission (FCC).

**Secondary outcomes:** Secondary outcomes are any additional outcomes that are to be addressed with the systematic review.

**Inclusion criteria:** History of attending to P&S in telehealth systems.

**Exclusion criteria:** No mention of security or privacy, HIPAA/HITECH compliance, information governance.

### SETTING

Inclusion and exclusion criteria for the setting and other relevant characteristics are provided below:

**Inclusion criteria**: Healthcare delivery in home, office, professional’s office, hospital, clinic, urban, and/or rural settings.

**Exclusion criteria:** Facilities that are not healthcare related or healthcare facilities that are not required to abide by HIPAA (e.g., those that do not bill electronically)

### SEARCH STRATEGIES

**Figure 1:**
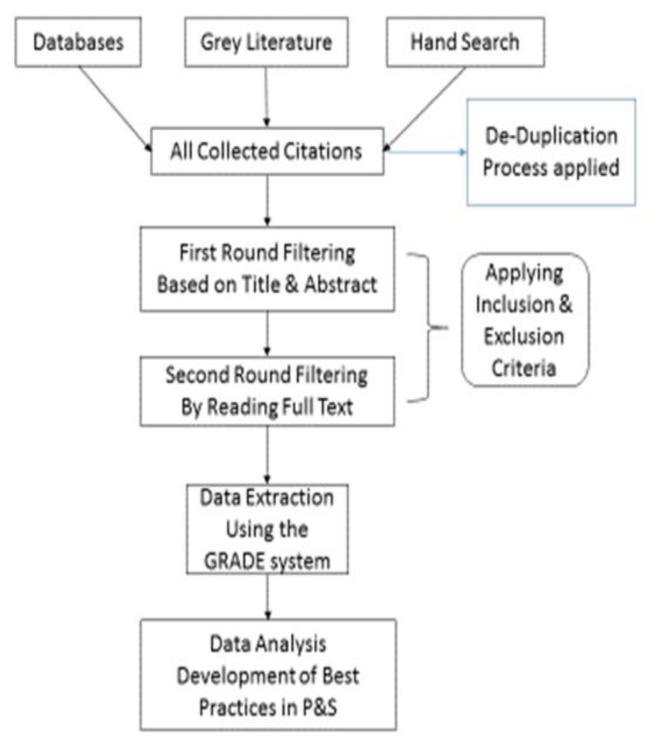
Flow chart to summarize search.

The studies to be evaluated in a systematic review can be found using the following methods:

- Search the bibliographic databases PubMed, EMBASE, Scopus, Compendex, and INSPEC.
- Search the Cochrane Library and the IEEE Xplore digital library.
- Search the Grey Literature, such as conference proceedings, research registries, and reports in databases and other suitable resources.
- Hand search appropriate journals to account for studies missed due to the imperfection of indexing, search strategies, and database compilation.
- Contact study authors and telehealth vendors as needed. The search strategy, executed by a reference librarian with expertise in the health sciences, is comprised of subject headings and keywords appropriate to the research question. These include synonyms for healthcare professionals, telehealth, healthcare delivery, and P&S. The only filter to be used during the search process is for the publication 2003 or later, as discussed earlier. All reference lists of selected articles are reviewed. Figure 1 presents a recommended search strategy.

### STUDY RECORDS

Export the search results into EndNote libraries. EndNote is a bibliographic management system that can organize references. The citations are to be de-duplicated using EndNote and the method outlined (Bramer et al, 2014).

The PDFs of the articles reviewed are to be stored in a shared Box account (i.e., a secure cloud storage tool in which users can share and send large documents as well as collaborate).

### HOW TO HANDLE DUPLICATE PUBLISHING

Reviews of duplicate publishing can be minimized by doing an author search and examining if the publications by the same authors are the same publications. If so, they are to be removed from the study records. When needed, study authors can be contacted to clarify whether or not the same dataset was used.

### SELECTION PROCESS

A process for selecting studies (such as using two independent reviewers) through each phase of the review is described.

Reviewers are to be blind to journals, study authors and institutions.

Two reviewers independently read the title and abstracts of the identified articles and determine eligibility based on the specified inclusion/exclusion criteria. Any disagreements between the reviewers are resolved by a third reviewer.

Inter-rater reliability is measured using kappa statistics. An inter-rater Kappa score is assessed during the inclusion/exclusion phase of review, to ensure a Kappa score at or above 0.8 as measured by Cohen’s Kappa (k) statistical test.

If the measure falls below a threshold for high correspondence (0.8), three reviewers discuss the selection until agreement is reached.

Full-text of items making this first cut are reviewed. Two reviewers screen these for inclusion/exclusion criteria. Additional information is sought from study authors when necessary. Selection disagreements are resolved through discussion; record reasons for excluding studies.

### DATA EXTRACTION PROCESS

The method of extracting data from reports (such as piloting forms, done independently, in duplicate) and any methods for obtaining and confirming data from investigators is described below.

Each publication meeting the inclusion criteria is reviewed and its characteristics documented using a standardized pre-tested data extraction form.

### DATA ITEMS

The data extraction form captures the following data items: descriptions about P&S, (confidentiality, integrity, availability, authentication, encryption, access control, physical security, policy, database backup, error detection, anti-virus, patches, robust system design, intrusion detection, safeguards), methods in each system, HIPAA compliance situations, study designs, settings and outcomes.

The reviewers will attempt to contact the authors of studies that are missing key data.

The reviewers will translate included studies written in Chinese or use online translation software. For any articles not in English nor Chinese, the reviewers will first use machine translation for the inclusion and exclusion criteria and when extracting specific data items attempt to find native speakers to assist with this process.

### RISK OF BIAS IN INDIVIDUAL STUDIES

To evaluate for the possibility of publication bias, the Peters test and a color-enhanced funnel plot done using STATA software will be used.

The methodological quality will be assessed using appropriate tools, including the Cochrane Collaboration’s Risk of Bias tool for randomized controlled trials (Higgins et al., 2011; Higgins & Green, 2011), the Cochrane Effective Practice and Organization of Care group’s tool for quasi-experimental designs, (EPOC, 2002) and the risk of bias tool developed in Waddington et al.’s (2012) study for regression-based studies (with special attention to confounding). Other observational studies will be assessed using the NOS score (Newcastle-Ottawa Quality Assessment Scale). The NOS score rates quality based on high risk (1–3 stars), medium risk (4–5 stars), or low risk (6–9 stars) NOS score (Wells et al., 2014).

### DATA SYNTHESIS

#### QUANTITATIVE ANALYSIS

The level of HIPAA compliance of each system included in the study can be quantified by ranking them according to their HIPAA compliance level. The HIPAA/HITECH audit protocol (United States Department of Health and Human Services, USDHHS, 2015) can be used as applicable to determine compliance within the identified systems. Some data may be analyzed but this may be limited due to the lack of quantifiable data in the privacy and security literature. Summary measures may include descriptive statistics (frequencies, percentages, measures of central tendency and variation). Data from different studies will be examined by levels of consistency using appropriate statistical tests.

#### QUALITATIVE ANALYSIS

If subgroups are available, researchers can choose some subgroups and subsets with similar characteristics and perform more in-depth comparisons among them. If available, researchers can compare the specific technologies each system used and compare their advantages and disadvantages in relation to P&S and HIPAA/HITECH compliance. Comparative content analysis (CCA) can be employed to determine themes across the qualitative data. This can be enhanced by using NVivo software which assists in the CCA and is excellent software to use to organize and analyze qualitative data.

### CONFIDENCE IN CUMULATIVE ESTIMATE

The strength of the body of evidence will be assessed using the GRADE (Grading of Recommendations, Assessment, Development and Evaluation) system (GRADE, 2015). This resource was developed in 2000 and is used to judge the quality of evidence in healthcare literature and evidence-based research. There are several different systems that are used in healthcare to evaluate literature. The GRADE system uses consistent and reliable criteria for systematic reviews. Quality of evidence criteria for systematic reviews includes:

1. Risk of bias/study limitations
2. Directness
3. Consistency of results
4. Precision
5. Publication bias

Researchers can assess the overall quality of evidence for every important outcome using the GRADE four-point ranked scale: (4) High; (3) Moderate; (2) Low; (1) Very low.

Narrative summaries are used as evidence for decisions about the quality of evidence and the strength of recommendations. Full evidence profiles suggested by the GRADE working group can be used and are based on systematic reviews. The evidence assessed and the methods used to identify and rank that evidence will be clearly described, such as reasons for up and down grading. Explicit consideration should be given to each of the GRADE criteria when assessing the strength of the recommendation (balance of desirable and undesirable consequences, quality of evidence, values and preferences, resource use) and a general approach as to how we dealt with those issues will be explained and reported.

The strength of recommendations will be explained using a two-point scale: (1) Weak/conditional; (2) Strong.

Definitions for each category should be consistent with those used by the GRADE Working Group, and decisions based on the strength of the recommendations transparently reported.

### LIMITATIONS

Reviewer bias may occur when reviewing the different manuscripts for inclusion into the systematic review since it includes judgement and opinions by the three reviewers.

### DISSEMINATION

The results of systematic reviews can be published in peer-reviewed journals, published on a grant website, and presented at conferences as appropriate.

## CONCLUSION

P&S concepts can be challenging for healthcare professionals to keep at the forefront when providing direct care to clients. While HIPAA/HITECH regulations provide guidance for healthcare professionals for in-person care, these regulations are extensive, may be different than state regulations, and can change.

Maintaining P&S for telehealth technologies can be even more daunting. These regulations are not always clear and may not cover every type of telehealth technology.

Therefore, it is important to provide healthcare professionals who employ telehealth technologies with best practices in P&S. This can not only enhance the P&S of their own practices, but enable them to feel more confident when discussing these issues with clients.

By sharing our systematic review protocol, we aim to provide other investigators with a methodology to conduct systematic reviews in an area of telehealth services. The development of the best practice guidelines can provide People with Disabilities (PwDs) with awareness of P&S risks, so that they can best manage those risks while still utilizing the benefits of the different telehealth technologies in their day-to-day lives.
